# Differences in drug use behaviors that impact overdose risk among individuals who do and do not use fentanyl test strips for drug checking

**DOI:** 10.1186/s12954-023-00767-0

**Published:** 2023-03-28

**Authors:** Alyssa Shell Tilhou, Jen Zaborek, Amelia Baltes, Elizabeth Salisbury-Afshar, Julia Malicki, Randall Brown

**Affiliations:** 1 Department of Family Medicine, Boston Medical Center, Boston University, 771 Albany St.Dowling 5 South, Rm 5507A, Boston, MA 02118 USA; 2 Department of Biostatistics & Medical Informatics, School of Medicine and Public Health, University of Wisconsin, Madison, WI USA; 3 Department of Family Medicine and Community Health, School of Medicine and Public Health, University of Wisconsin, Madison, WI USA

**Keywords:** Fentanyl test strip (FTS), Opioids, Harm reduction, Drug testing, Overdose risk

## Abstract

**Background:**

Opioid-involved overdose continues to rise, largely explained by fentanyl adulteration of the illicit opioid supply. Fentanyl test strips are a novel drug checking tool that can be used by people who use drugs to detect the presence of fentanyl in drug products. However, it is unclear whether fentanyl test strip use can prompt behavior changes that impact risk of overdose.

**Methods:**

In this mixed-methods study involving a structured survey (*n* = 341) of syringe service program clients in southern Wisconsin, we examined the association between fentanyl test strip use and overdose risk behaviors in scenarios where the presence of fentanyl is confirmed and unknown. Individual items were transformed into summary scales representing the performance of riskier and safer behaviors. Linear regression examined the association of behaviors with FTS use. Models are adjusted for study site, race/ethnicity, age, gender, drug of choice, indicator of polysubstance use, times used per day, and lifetime overdose count.

**Results:**

In response to survey questions before prompting about fentanyl risk, people who used fentanyl test strips reported an increased number of safer (*p* = 0.001) as well as riskier behaviors (*p* = 0.018) relative to people who did not use fentanyl test strips. The same held true in situations when fentanyl adulteration was suspected, though fentanyl test strip use lost significance in the fully adjusted model examining safer behaviors (safer: *p* = 0.143; riskier: *p* = 0.004). Among people who use fentanyl test strips, in unadjusted models, a positive test result was associated with more safer behaviors and fewer riskier behaviors, but these associations became nonsignificant in fully adjusted models (safer: *p* = 0.998; riskier: *p* = 0.171). Loss of significance was largely due to the addition of either polysubstance use or age to the model.

**Conclusions:**

Fentanyl test strip use is associated with behaviors that may impact overdose risk, including safer and riskier behaviors. Specifically, a positive test result may promote more risk reducing behaviors and fewer risk enhancing behaviors than a negative test result. Results suggest that while FTS may promote safer drug use behaviors, outreach and education should emphasize the need for multiple harm reduction techniques in all scenarios.

**Supplementary Information:**

The online version contains supplementary material available at 10.1186/s12954-023-00767-0.

## Background

Rates of opioid-involved overdose continue to rise annually. In 2020, opioid-involved overdose deaths reached a record high of 70,000 [1], and preliminary analyses suggest this number rose another 15% in 2021 [2]. This persistent upward climb is largely explained by adulteration of the illicit drug supply with fentanyl and other chemically related synthetic opioids. As a result of this undisclosed adulteration in an unregulated drug market, people who use drugs (PWUD) often do not know if their drugs contain fentanyl. Rising rates of co-involved stimulant-fentanyl overdose [3, 4], particularly among individuals without known opioid use disorder [5], demonstrate the substantial risk of overdose from unintentional fentanyl use.

Drug checking, a harm reduction intervention that allows PWUD to assess the chemical composition of their drug samples, has expanded in recent years in response to the synthetic opioid overdose crisis [6]. A variety of drug checking services have emerged including handheld mass spectrometry, infrared spectroscopy, and immunoassays in the form of fentanyl test strips (FTS), among others [7, 8]. Of these technologies, FTS may represent a uniquely scalable harm reduction tool to counter rising fentanyl overdose. FTS are small immunoassay test kits originally designed for urine drug testing that have been repurposed off label for direct testing for fentanyl and select analogues in drug residue solution [9–11]. As an affordable and transportable technology, FTS allow PWUD to assess their drug samples for the presence of fentanyl without synchronous attendance by a trained professional. As a result, FTS offer the advantage of being independently used by PWUD in community settings.

Thus far, studies investigating the feasibility and acceptability of FTS among PWUD have yielded promising results [12, 13]. In addition, research has demonstrated that FTS use is associated with using less drug per dose, using the drug more slowly, using test doses, using with someone else, and having naloxone (the opioid overdose antidote) [6, 9, 14–19]. Despite promising early findings, key questions remain about the effectiveness and utility of FTS as an opioid overdose harm reduction tool. First, existing literature does not confirm whether FTS use changes the behaviors of PWUD. Observed associations could, instead, reflect a selection bias such that people inclined toward harm reduction behaviors are more likely to report using FTS. Second, it is unclear whether FTS can continue to motivate safer behavior in a market highly saturated in fentanyl thereby yielding repeated positive test results [16]. Third, FTS can produce false negatives due to concentrations below the detection threshold [8, 11], the presence of undetectable analogs [8, 11], or inaccurate test preparation or interpretation [20]. If negative test results promote riskier behaviors, false negatives could increase risk for overdose, particularly among individuals unintentionally using opioids with low opioid tolerance.

To better understand the impact of FTS use on drug use behaviors that impact overdose risk, we compare the behaviors reported by PWUD who do and do not use FTS. Among PWUD who report using FTS, we also compare reported behaviors when FTS results are positive and negative, and when they do not have FTS. Findings are based on a sample of PWUD with high rates of seeking fentanyl, and high penetration of fentanyl in their drug supply [20]. In this way, this study aims to investigate the degree to which FTS use may promote drug use behaviors that impact risk of opioid overdose (herein, “overdose risk behaviors”).

## Methods

### Design

Data for this study come from Screening for Adulterants like Fentanyl and Risks of Fentanyl Test Strip Use (SAFeR), a sequential exploratory mixed-methods study on overdose risk behaviors among people who do and do not use FTS to test drugs for fentanyl. Phase 1 involved completion of semi-structured, open-ended interviews. Content and language from Phase 1 contributed to a survey focused on FTS use and related overdose risk behaviors in a separate, larger sample. The study protocol was approved by the Institutional Review Board at the University of Wisconsin School of Medicine and Public Health (UWSMPH). SAFeR was funded by an internal grant through the Department of Family Medicine and Community Health at UWSMPH. For additional details about SAFeR, please see previously published work [20].

### Data collection

The SAFeR Phase 2 survey was fielded in March through September of 2021. All recruitment and survey completion occurred at four sites of a syringe service program (SSP) in southern Wisconsin (Beloit, Kenosha, Madison, and Milwaukee) through flyers and verbal advertisement. At the time of data collection, FTS were only available through this SSP. Inclusion criteria required age ≥ 18 years, prior receipt of client services at the SSP site, at least 3 cumulative months of weekly nonprescribed substance use (excluding cannabis) as well as weekly use over the past 30 days, ability to speak and read in English, and ability to navigate a tablet-based survey. Exclusion criteria included previous SAFeR participation and impaired cognition or safety concerns due to intoxication. Respondents provided digital consent within the survey. Respondents received $15 at completion or $5 after failing inclusion/exclusion criteria.

Survey questions asked respondents about sociodemographic characteristics, substance use treatment utilization, characteristics of FTS use, perceptions of fentanyl and overdose risk, and overdose risk behaviors including in relation to using FTS. The list of risk behaviors was based on Phase 1 interviews, which aimed to identify a comprehensive set of behaviors reported by PWUD that they perceive as increasing or decreasing risk of overdose. Free-listing was used to tabulate and identify the most salient behaviors for incorporation into the survey instrument [21]. Surveys were self-administered on a tablet computer, which automatically stored and then transmitted data using Research Electronic Data Capture (REDCap), electronic data capture tools hosted at the UWSMPH Department of Family Medicine and Community Health. REDCap is a secure, web-based software platform designed to support data capture for research studies [22, 23]. In total, SAFeR collected 341 surveys.

### Community engagement

To enhance the alignment of study goals and content with the priorities of community members, SAFeR engaged several external groups in the development of study materials. First, the study team sought feedback from SSP staff and leadership on the study protocol, recruitment strategy and study materials. In addition, SAFeR worked with a community-based research consultation group (Community Advisors on Research Design and Strategies (CARDS)) to receive feedback on the content and wording of Phase 2 survey items [24] (Please see prior published work for details [20]). Finally, the study team received technical assistance on the design of the survey questionnaire from the University of Wisconsin Survey Center.

### Measures

For a full list of measures and characteristics of the sample, see previously published material[20]. To facilitate interpretation of study findings, we again report the following individual characteristics and FTS use characteristics: *individual characteristics:* study site, age, gender (man, woman, nonbinary, trans, and other), race and ethnicity (American Indian or Alaskan Native, Asian, Black or African American, Hispanic or Latinx, Native Hawaiian or Other Pacific Islander, White, and other), education, unemployment (defined as reporting no employment while looking for a job), years of use, drug of choice, usual route of drug use, drug use frequency, polysubstance use, and lifetime overdose count; *FTS use characteristics:* count of FTS use, frequency of FTS use, frequency of positive test results, and drugs tested with FTS.

To understand how the relationship between FTS use and overdose risk behaviors, respondents were asked about a set of overdose risk behaviors that emerged in Phase 1 interviews. These behaviors were presented in four scenarios: (1) “generally when using your drug of choice” (“unspecified scenarios”) (2) “when you think your drugs contain fentanyl but you do not have a fentanyl test strip;” (3) “when a test strip shows there is fentanyl;” and (4) “when a test strip shows there is no fentanyl in your drugs.” In all scenarios, respondents were asked how often they perform the following behaviors (1 = never, rare, sometimes, most of the time, 5 = every time you use). Riskier behaviors included: use enough to get high, use enough to nod out, use more when you have Narcan, use alone, and mix your drug of choice with other drugs or alcohol. Safer behaviors included: do a test dose, use enough to stop withdrawing but not to get high, call someone before using if you are alone, let someone else use first if you are with others, and make sure you have Narcan in case someone needs it. To understand overdose risk behaviors specifically in the context of fentanyl or FTS, five additional behaviors were asked in scenarios 2–4. These behaviors included three safer behaviors (“do not use it”, “use less than normal”, and “snort or shoot into the muscle instead of inject into the vein”) and two riskier behaviors (“use more than normal” and “inject into the vein instead of snort or shoot in the muscle”).

### Analysis

Demographic characteristics and FTS use characteristics were summarized for the sample. Responses to items on overdose risk behaviors were transformed into summary scores. Summary scores were calculated as the average Likert response to those behavior questions. A total of eight summary scores–one for each set of riskier and safer behaviors in each of the four scenarios—were used as outcomes for analysis. Cronbach’s alpha was calculated for each summary score overall and by FTS use when applicable. In addition, the reliability with each scale item dropped was examined.

For the general and suspected fentanyl scenarios, unadjusted and adjusted linear regression models were fit for each outcome to examine the relationship between riskier and safer behaviors and FTS use. For positive and negative FTS result scenarios, unadjusted and adjusted linear regression models were fit for each outcome to examine the relationship between riskier and safer behaviors with the FTS result. Adjusted models included covariates of site, race/ethnicity, age, gender, drug of choice, indicator of polysubstance use, times used per day, and lifetime overdose count. Mean differences, 95% confidence intervals, and p-values are reported. Significance was assessed at the alpha = 0.05 level and no adjustments were made to account for inflated type 1 error rate. We present the results of adjusted models. Please see Additional file 1: Appendix 1 for unadjusted linear regression results for individual scale items.

Statistical analysis was conducted using R version 4.1.3 (2022-03-10) with the psych package used for calculating Cronbach’s alpha [25].

## Results

### Sociodemographic and FTS use characteristics

Drug use characteristics of the sample (*n* = 341) are presented in Table 1. The most common drug of choice was heroin (70.7%), the most common usual route of drug use was intravenous (87.9%), and 73.9% reported polysubstance use. Average lifetime number of overdoses was 4.4 (sd = 8.4). Most respondents (*n* = 274; 80.4%) reported using FTS themselves or using drugs tested with FTS by someone else. Sociodemographic characteristics for the sample have been published in detail previously. The sample was 59.6% men and reported a mean age of 35.7 years. The majority of respondents reported unemployment in the past six months (71.5%) and identified as non-Hispanic white (77.4%). Remaining racial and ethnic demographics included: 7.1% non-Hispanic Black or African American, 6.2% Hispanic, and 2.9% non-Hispanic American Indian with an additional 1.8% and 4.7% reporting other or multiple categories, respectively. Overall, educational attainment was low with 3.2% reporting completion of a 4-year college degree and 11.2% reporting less than a high school degree. Each site contributed roughly 20–30% of respondents.

**Table 1:** Characteristics of syringe service clients in southern Wisconsin with respect to FTS use

Characteristics	All	FTS use	No FTS use
	N = 341	N = 274	N = 67
	n(%) or m(sd)		
Current drug of choice			
Heroin	241 (70.7)	202 (73.7)	39 (58.2)
Fentanyl	43 (12.6)	36 (13.1)	7 (10.4)
Opioid analgesics	10 (2.9)	2 (0.7)	8 (11.9)
Cocaine or Crack Cocaine	32 (9.4)	23 (8.4)	9 (13.4)
Methamphetamine	15 (4.4)	11 (4.0)	4 (6.0)
Synthetics^1^	0 (0.0)	0 (0.0)	0 (0.0)
Prescription anxiety drugs	0 (0.0)	0 (0.0)	0 (0.0)
Prescription amphetamines	0 (0.0)	0 (0.0)	0 (0.0)
Some other drug	0 (0.0)	0 (0.0)	0 (0.0)
Usual route of drug use			
Inject or shoot it into a vein	299 (87.9)	255 (93.4)	44 (65.7)
Inject or shoot it into a muscle	4 (1.2)	2 (0.7)	2 (3.0)
Snort it	24 (7.1)	10 (3.7)	14 (20.9)
Smoke it	12 (3.5)	6 (2.2)	6 (9.0)
Eat or swallow it	1 (0.3)	0 (0.0)	1 (1.5)
Insert it rectally	0 (0.0)	0 (0.0)	0 (0.0)
Use skin popping	0 (0.0)	0 (0.0)	0 (0.0)
Some other method	0 (0.0)	0 (0.0)	0 (0.0)
Drug of choice use frequency in past 30 days			
Less than once per day	18 (5.3)	12 (4.4)	6 (9.0)
1 time per day	15 (4.4)	10 (3.6)	5 (7.5)
2 times per day	40 (11.7)	30 (10.9)	10 (14.9)
3 or more times per day	268 (78.6)	222 (81.0)	46 (68.7)
Polysubstance use^2^	252 (73.9)	210 (76.6)	42 (62.7)
Years of drug use	12.2 (8.2)	11.9 (7.9)	13.0 (9.4)
Lifetime count of opioid overdose	4.4 (8.4)	4.7 (9.1)	3.3 (4.5)

Abbreviations: FTS, fentanyl test strips; m: mean; sd: standard deviation; adj: adjusted; unadj: unadjusted; GED: General Educational Development

Missing: Gender – 2; Race and Ethnicity – 1; Education – 1; Employment – 1; Years of drug use – 3; Current drug of choice – 1; Usual route – 1; Overdose count – 4;

Associations with continuous variables were tested using Mann–Whitney–Wilcoxon tests and associations with categorical variables were tested with chi-square tests

^1^Synthetics refers to synthetic opioids such as U47700, U4 or “Pink.”

^2^Polysubstance use is defined as use of more than one category of drugs excluding alcohol and marijuana

Respondents who reported FTS use received additional questions about using FTS. Details have been published previously [20]. Over one-third reported using FTS most of the time or more with their drug of choice. Reported drugs tested with FTS included cocaine (27.0%), crack cocaine (32.1%), fentanyl (36.9%), heroin (92.7%), cannabis (4.7%), methamphetamine (19.0%), opioid analgesics (11.3%), and stimulant medications (1.1%).

### Behaviors in unspecified scenarios

Analyses about overdose risk behaviors “generally when using your drug of choice” are shown in Table 2. Responses about safer behaviors exhibited an overall Cronbach alpha of 0.70 overall (range 0.61–0.73 on individual items). Adjusted and unadjusted models demonstrate that people who use FTS perform more safer behaviors than people who do not use FTS. Differences in mean scores between FTS users and nonusers were less than 1 on all items, indicating a difference less than consecutive choices on the Likert scale. Responses about riskier behaviors exhibited an overall Cronbach’s alpha of 0.56 (range 0.40–0.60 on individual items). People who use FTS reported performing more riskier behaviors than people who do not use FTS in adjusted and unadjusted models. Again, the difference in mean scores between the two groups was less than 1.

**Table 2:** Results from linear regression examining the association between FTS use and overdose risk behaviors in unspecified and suspected adulteration scenarios

		Unadjusted			Adjusted^b^		
Scenario		Coefficient^a^	95% CI	*p* Value	Coefficient^a^	95% CI	*p* Value
Unspecified	Safer	0.42	0.22 to 0.62	< 0.001	0.34	0.13 to 0.55	0.001
	Riskier	0.21	0.04 to 0.37	0.015	0.20	0.04 to 0.37	0.018
Suspected	Safer	0.20	0.02 to 0.39	0.033	0.14	− 0.05 to 0.33	0.143
	Riskier	0.32	0.14 to 0.50	< 0.001	0.27	0.09 to 0.46	0.004

Abbreviations: FTS, fentanyl test strip. CI; confidence interval

^a^Coefficients of FTS use indicator. Positive values indicate that FTS users perform the behavior more often than non-FTS users

^b^Adjusted models include indicators for site, race/ethnicity, age, gender, drug of choice, indicator of polysubstance use, times used per day, and lifetime overdose count

### Behaviors when fentanyl adulteration suspected

Analyses about overdose risk behaviors when “you think your drugs contain fentanyl but you do not have a test strip” are shown in Table 2. Responses about safer behaviors exhibited an overall Cronbach’s alpha of 0.73 (range 0.64–0.75 on individual items). In the unadjusted model, FTS use was associated with performing more safer behaviors. However, in the fully adjusted model, FTS use lost significance. The significant association of FTS use with safer behaviors was eliminated by adjusting individually for either overdose count, SSP site, or gender (not shown). Responses about riskier behaviors exhibited an overall Cronbach’s alpha of 0.71 (range 0.62–0.71 on individual items). In unadjusted and adjusted models, FTS use was associated with performance of more riskier behaviors.

### Behaviors with a positive or negative FTS Result

Items about FTS results were only presented to respondents who reported prior FTS use. Analyses about overdose risk behaviors when “a test strip shows there is fentanyl in your drugs” is presented in Table 3. Responses about safer behaviors when the FTS result is positive exhibited an overall Cronbach’s alpha of 0.73 (0.68–0.72 on individual items) for safer behaviors and 0.70 (0.62–0.73 on individual items) for riskier behaviors. With a negative test result, responses exhibited an overall Cronbach’s alpha of 0.73 (0.69–0.75 on individual items) for safer behaviors and 0.71 (0.64–0.71 on individual items) for riskier behaviors. A positive FTS result was associated with performing more safer behaviors among FTS users than a negative FTS result. However, this effect became nonsignificant in the fully adjusted model. Examining individual covariates suggested that this effect was insignificant after adjusting for either polysubstance use or age (see Additional file 1: Appendix 2 for details). With respect to riskier behaviors, a positive FTS result was associated with performing fewer riskier behaviors in unadjusted models, but this association becomes nonsignificant in fully adjusted models. Examination of models with individual covariates indicates this effect is insignificant after adjusting for age.

**Table 3:** Results from linear regression examining the association between FTS use and overdose risk behaviors when FTS results are positive relative to negative

	Unadjusted			Adjusted^b^		
	Coefficient^a^	95% CI	*p* Value	Coefficient^a^	95% CI	*p* Value
Safer	0.20	0.14 to 0.25	< 0.001	− 0.00	− 0.36 to 0.36	0.998
Riskier	− 0.19	− 0.25 to − 0.13	< 0.001	− 0.27	− 0.64 to 0.11	0.171

Abbreviations: FTS, fentanyl test strip. CI; confidence interval

^a^Coefficients of difference between positive and negative behaviors. Positive values indicate that FTS users perform the type of behavior more often when there is a positive FTS result compared to a negative result

^b^Adjusted models include indicators for site, race/ethnicity, age, gender, drug of choice, indicator of polysubstance use, times used per day, and lifetime overdose count

## Discussion

In this study of SSP clients in southern Wisconsin, we find that PWUD who use FTS perform more drug use behaviors that are both safer and riskier for overdose compared with PWUD who do not use FTS. This pattern persists even in scenarios when fentanyl adulteration is suspected. While the integration of both safer and riskier behaviors may seem counterintuitive, their coincidence could reflect that PWUD who use FTS have strong motivation in addition to heightened challenges to behavior change, perhaps in the setting of longer use history, higher use frequency or greater addiction severity relative to PWUD who do not use FTS. Addiction is the compulsive use of substances at quantities beyond one’s control despite the harmful negative consequences caused by substance use [26]. Thus, the core features of addiction could drive the performance of riskier as well as safer behaviors as individuals attempt to minimize the harms of their addiction. From another perspective, people who perform riskier behaviors may recognize those risks and therefore intentionally use FTS along with other risk reducing behaviors to stay as safe as possible. Prior analyses from SAFeR suggested an association of FTS use with riskier behaviors and/or higher addiction severity such as higher frequency of drug use, injection use, report of seeking fentanyl, and polysubstance use.

These findings of coincident safer and riskier behaviors speak to a long-standing concern about the propensity of harm reduction tools to promote compensatory drug use behaviors [27]. In this cross-sectional analysis, we cannot rule out that FTS use prompted riskier drug use behaviors. However, these results poignantly demonstrate that individuals who perform riskier behaviors do not want to overdose and do perform safer behaviors, as well. Moreover, the performance of both riskier and safer behaviors suggests against the likelihood that people who use FTS are simply safer in general. This circumstantial evidence against a selection bias among people who use FTS upholds the possibility that FTS do prompt safer behaviors. In support, prior analyses with the same sample demonstrated no differences in treatment seeking behaviors by FTS use except with respect to recent methadone use [20].

As for the impact of FTS results on overdose risk behaviors, we find that a positive FTS result is associated with more safer behaviors and fewer riskier behaviors than a negative FTS result. However, these associations became nonsignificant after adjusting for polysubstance use and age, which may suggest a role for these factors in influencing drug use behaviors. Notably, these findings emerge in a sample reporting frequent positive test results [20], which suggests that FTS may promote safer behaviors even in the context of high fentanyl penetration and predictably positive FTS results such as in Wisconsin [28]. Future analyses should consider exploring the psychosocial meaning of using FTS among individuals for whom results are usually positive.

Alongside the direct relationship between positive FTS test results and safer behaviors, we observed that negative FTS test results were associated with fewer safer behaviors. While logical, these findings raise safety concerns given risks of false negatives, especially for fentanyl analogs [11, 29]. These risks are particularly critical for people who primarily use stimulants and other non-opioid drugs and therefore have lower physiologic tolerance to high potency opioids [5]. Thus, it is critical that harm reduction practitioners counsel PWUD to implement multiple overdose risk reduction techniques regardless of FTS results. The potential for false negatives to promote reduced caution urgently compels for wider access to robust drug checking services and supervised consumption services [7, 30].

This analysis had several limitations. First, this study’s cross-sectional design limits our ability to evaluate causal relationships between FTS use and drug use behaviors. Second, we collected no identifying information to maximize privacy protections for respondents. As a result, we cannot guarantee that respondents did not participate more than once. Third, our sample is limited to SSP clients in southern Wisconsin and therefore may not reflect the experiences of PWUD who do not use syringe services or who reside in other regions. Fourth, we did not use a validated scale to rate behaviors with respect to risk of overdose. Instead, we used the behaviors perceived by respondents (in Phase 1 of SAFeR) as impacting overdose risk. Thus, behavior scales were based on rigorous qualitative methods and exhibited high internal consistency via Cronbach alpha scores.

## Conclusions

In sum, we find that FTS use is associated with performing more safer and more riskier drug use behaviors with respect to overdose. In addition, we find that positive FTS results are associated with more safer behaviors and fewer riskier behaviors than negative test results. These findings suggest that FTS may help promote behaviors to reduce overdose risk among PWUD even when fentanyl penetration is high and use of fentanyl common. Moreover, these findings demonstrate that the vast majority of PWUD want to be safer and do not want to overdose. Further innovation of harm reduction strategies is urgently needed to support PWUD gain further control over their health and well-being. Despite promising indicators that FTS promote overdose risk reducing behaviors, health care providers should emphasize the performance of overdose risk reducing behaviors regardless of FTS results due to the risk of false negatives and the perpetual hazard of emerging adulterants in the context of an unregulated drug market. As the drug supply evolves, further research will be needed to evaluate the ongoing impact of FTS on drug use behaviors.

## Supplementary Information

**Additional file 1**. Appendices 1, 2.

## Data Availability

The datasets generated and analyzed during the current study are not publicly available at the time of publication.

## Abbreviations

FTS: Fentanyl test strips
PWUD: People who use drugs
SSP: Syringe service program
SAFeR: Screening for Adulterants like Fentanyl and Risks of Fentanyl Test Strip Use
UWSMPH: University of Wisconsin School of Medicine and Public Health
DOC: Drug of choice
