# Supplementary material for: Differences in drug use behaviors that impact overdose risk among individuals who do and do not use fentanyl test strips for drug checking
Source: Harm Reduct J. 2023 Mar 28;20:41. doi: 10.1186/s12954-023-00767-0 (PMC10053743; doi:10.1186/s12954-023-00767-0)
Supplement: Supplementary file 1 — Additional file 1. Appendices 1, 2. [file 12954_2023_767_MOESM1_ESM.docx]

**Appendix 2. Full results from linear regression examining the association between FTS use and overdose risk** **behaviors when FTS results are positive relative to negative**

|  | **Safer** | | | **Riskier** | | |
| --- | --- | --- | --- | --- | --- | --- |
|  | Coefficient^a^ | 95% CI | p-value | Coefficient^a^ | 95% CI | p-value |
| Unadjusted | 0.20 | 0.14 to 0.25 | < 0.001 | -0.19 | -0.25 to -0.13 | < 0.001 |
| Adjusted by: |  |  |  |  |  |  |
| All covariates | -0.00 | -0.36 to 0.36 | 0.998 | -0.27 | -0.64 to 0.11 | 0.171 |
| Site | 0.14 | 0.02 to 0.27 | 0.028 | -0.19 | -0.32 to -0.06 | 0.006 |
| Race/ethnicity | 0.13 | 0.01 to 0.26 | 0.041 | -0.28 | -0.42 to -0.15 | < 0.001 |
| Age | 0.11 | -0.15 to 0.38 | 0.395 | -0.19 | -0.47 to 0.10 | 0.201 |
| Gender | 0.22 | 0.15 to 0.30 | < 0.011 | -0.20 | -0.28 to -0.12 | < 0.001 |
| Drug of choice | 0.20 | 0.14 to 0.27 | < 0.001 | -0.19 | -0.26 to -0.12 | < 0.001 |
| Polysubstance use | 0.07 | -0.05 to 0.20 | 0.256 | -0.16 | -0.30 to -0.03 | 0.020 |
| Frequency of use | 0.21 | 0.14 to 0.27 | < 0.001 | -0.20 | -0.27 to -0.14 | < 0.001 |
| Overdose times | 0.19 | 0.12 to 0.25 | < 0.001 | -0.17 | -0.24 to -0.10 | < 0.001 |

^a^Coefficients of difference between positive and negative behaviors. Positive values indicate that FTS users perform the type of behavior more often when there is a positive FTS result compared to a negative result.
